# The effects of food level and social density on reproduction in the Least Killifish, *Heterandria formosa*

**DOI:** 10.1002/ece3.4634

**Published:** 2018-12-14

**Authors:** Kathryn N. Leatherbury, Joseph Travis

**Affiliations:** ^1^ Department of Biological Science Florida State University Tallahassee Florida

**Keywords:** crowding, food limitation, population density, reproduction, social density, social stress

## Abstract

The feedbacks from population density to demographic parameters, which drive population regulation, are the accumulated results of several ecological processes. The compensatory feedback from increased population density to fertility includes at least two distinct factors, the effects of decreases in per capita food level and increases in the social density (the number of interacting individuals). Because these effects have been studied separately, their relative importance is unknown. It is also unclear whether food limitation and social density combine additively to influence fertility. We investigated these questions with two factorial experiments on reproduction in the Least Killifish, *Heterandria formosa*. In one experiment, we crossed two levels of density with two levels of a total food ration that was distributed to all individuals. In the other experiment, we crossed two levels of density with two levels of per capita food. Whereas the first experiment suggested that the effects of variation in food level and density were synergistic, the second experiment indicated that they were not. The apparent synergism—the statistical interaction of food and density levels—was the result of confounding per capita food with social density in that design. In the second experiment, the effects of social density on reproductive rate were stronger than the effects of food level, whereas the effects of food level were stronger on offspring size at parturition than those of social density. The results suggest that the social stresses that emerge at higher densities play an important role in the compensatory response of fertility to density, a role, that is, at least as important as that of decreased per capita food levels.

## 1 INTRODUCTION

Feedbacks from population density to demographic parameters like survival and fertility rates are the fundamental drivers of population regulation (Herrando‐Pérez, Delean, Brook, & Bradshaw, 2012). These feedbacks are statistical summaries of many ecological processes that can act in natural populations. For example, depensatory feedback on survival can accumulate from the effects of multiple predators and pathogens (Korpimäki, 1993) and compensatory feedback on fertility can accumulate from food limitation and predator‐induced stress (Krebs, 2011). The various ecological processes producing first‐ and second‐order feedbacks can either oppose or reinforce one another (Bassar et al., 2012; Kolb, Dahlgren, & Ehrlén, 2010) and, in that light, it is important to discern their various roles in different circumstances (Boonstra & Krebs, 2012).

This is especially so for compensatory effects on fertility, which can emerge from a variety of sources in different systems with different implications for numerical dynamics. For example, compensatory feedback through territory limitation (Treinys, Bergmanis, & Väli, 2016) or nesting sites (Brazill‐Boast, Pryke, & Griffith, 2013), in which there are always a finite number of successfully reproducing individuals, provokes contest competition that can be less potentially destabilizing than compensatory feedback through scramble competition for limited food (Lomnicki, 1988; Pinot et al., 2016).

The relative importance of several candidate processes for creating compensatory feedback on fertility remains unclear. In particular, the relative contributions of food limitation and the inhibition of reproduction through the social stress of crowding remain unexplored. To be sure, there have been extensive studies of each effect in small mammals (Batzli, 1977; Boonstra & Boag, 1992; Gauthier, Berteaux, Krebs, & Reid, 2009; Krebs, Lambin, & Wolff, 2007; Oksanen, Oksanen, Dahlgren, & Olofsson, 2008; Wolff, 1997) and aquatic invertebrates (Beserra, Freitas, Souza, Fernandes, & Santos, 2009; Brent, 2010; Burns, 1995; Carvalho & Hughes, 1983; Guisande, 1993; Hooper, Sibly, Hutchinson, & Maund, 2003; Rose, Rees, & Grubb, 2002; Yang, Hu, Shi, & Zhai, 2015). However, there have been few studies, even in these systems that were designed to compare the relative strength of the two effects.

That each process could be important is obvious, but it is unclear whether they exert equally strong influences. There is ample evidence in animals beyond small mammals and aquatic invertebrates that crowding itself, independent of food level, can elevate stress levels, inhibit fertility, or reduce somatic growth rates (Borg, Rowden, Attrill, Schembri, & Jones, 2006; Dettmer, Novak, Meyer, & Suomi, 2014; Doidge, Croxall, & Baker, 1984; Li, Dong, Lei, & Li, 2007; Ramsay et al., 2006; Steinwascher, 1979; Warren, 1973; Weber, Vallejo, Lankford, Silverstein, & Welch, 2011; Wilbur, 1977a). Similarly, there is ample evidence that food limitation can create strong compensatory feedback; this has been demonstrated in fishes, for example, by both experimental (Pollux & Reznick, 2011; Reznick, Callahan, & Llauredo, 1996; Travis, Farr, Henrich, & Cheong, 1987) and observational (Okamoto, Schmitt, Holbrook, & Reed, 2012) studies.

Few experimental studies have manipulated food level and density in a manner that avoided confounding food limitation with social density (here defined as the number of interacting individuals within a patch in nature or an experimental enclosure). Most ecological studies of how density affects vital rates, or life‐history traits associated with vital rates, either varied total density and distributed a constant total food ration to the individuals at each density or did not control the amount of food available (Brent, 2010; Dash & Hota, 1980; Forrester, Harmon, Helyer, Holden, & Karis, 2011; Weeks, 1993; Wilbur, 1977b). This design confounds per capita food levels and social density; the higher densities have less food per capita and more socially interacting individuals, so the two factors are acting together, making their relative contributions inseparable. The factorial experiments that have varied food level and density simultaneously have, for the most part, distributed, at each designated food level, constant total rations of food (Berven & Chadra, 1988; Laufer & Maneyro, 2008; Samhouri, 2009; Wilbur, 1977b). This design allows the main effect of food level variation to be estimated independently of density but not vice versa, because different densities still confound changes in per capita food availability with changes in the number of interacting individuals. While the results of this kind of factorial experiment can be examined in a *post hoc* fashion in terms of supply‐demand ratios in different treatment combinations (Wilbur, 1977b), this approach can diagnose only that a factor other than per capita food availability might be acting and cannot estimate its importance relative to food level variation.

There are several reasons to predict strong effects of social density independent of the effects of food level variation. First, as noted above for many animals, increased social density is known to increase stress levels. Second, experiments with cladocerans have shown that the effects of lower food levels on reproductive parameters are not always in the same direction as the effects of increased density (Guisande, 1993). Third, among the factorial experiments that have varied total food level and density, several have found strong statistical interactions, indicating that the effects of one factor depend upon specific levels of the other (Berven & Chadra, 1988; Burns, 1995; Wilbur, 1977a). Similarly, Rose et al. (2002) found different effects of density in two experimental manipulations conducted at separate food levels. Fourth, in aquatic animals, water conditions by different levels of crowding have been shown to exert compensatory effects on reproduction in four species of *Daphnia* (Burns, 1995) and on larval growth rate in tadpoles (Steinwascher, 1979).

In this paper, we present the results of two experiments on the Least Killifish, *Heterandria formosa*, which were designed to examine the contributions of food level variation and variation in social density on reproduction. In the first experiment, we used a traditional ecological design that crossed two food levels with two density levels but, at each food level, we distributed the food as a total ration to all individuals. In the second experiment, we used a factorial design with two levels of per capita food and two levels of density. Our second experiment allows the contributions of food level to be separated from those of social density. The comparison of the two experiments allows us to assess the ways in which the results of the experiment using total food ration might prove misleading about the relative contributions of food limitation and social density. This approach is comparable to that of Beserra et al. (2009), in which variation in the density of larval *Aedes aegypti* was crossed with two food distribution treatments, one in which the same total food was offered to each experimental unit and one in which the same per capita food levels were offered to each experimental unit (food levels were not varied).

## 2 MATERIALS AND METHODS

### 2.1 The Least Killifish

**Figure 1:**
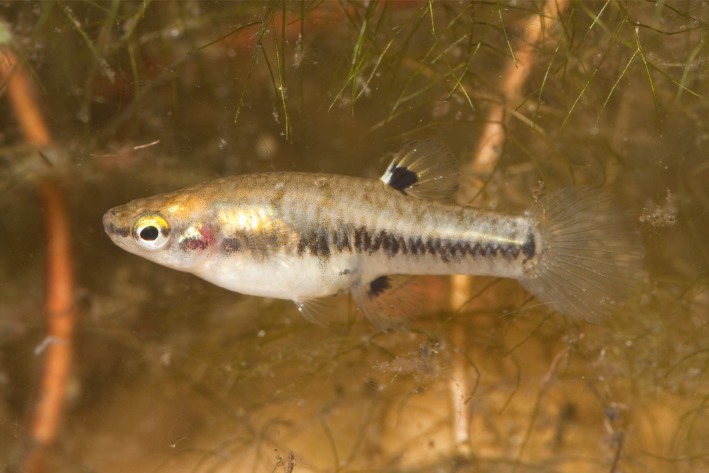
Female *Heterandria formosa*, photo courtesy of Pierson Hill

*Heterandria formosa* is a small poeciliid fish native to the coastal plain of the southeastern United States (Figure 1). Females range in standard length (tip of snout to base of tail) from 15–25 mm and males range from 13–19 mm. Populations of *H. formosa* persist in a range of habitats from acidic, lentic ponds with high predator densities to basic, lotic spring‐fed rivers with lower predator densities (Leips & Travis, 1999; MacRae & Travis, 2014). Reproduction is matrotrophic; unfertilized ova have very little yolk and nearly all of the energy for development is provided continuously after fertilization via transfer from mother to embryo through a placenta (Schrader & Travis, 2012). Embryos increase in dry mass during development by 30‐ to 50‐fold. Females provision several broods simultaneously, a phenomenon known as superfetation (Travis et al., 1987). Previous experiments have shown that reproductive traits are sensitive to variation in food level (Reznick et al., 1996; Travis et al., 1987) and density (Leips, Travis, & Rodd, 2000) and social density (Leips, Richardson, Rodd, & Travis, 2009), but no experiments have varied food and density simultaneously. The gestation period is 25–28 days, which means experimental manipulations of food and density on wild‐caught females must be done for at least 2 months. Offspring born in the first month of an experiment will have been influenced by the manipulation only late in gestation, whereas offspring born in the second month will have been influenced by the manipulation for their full gestation period. Therefore, we report only results from the second month.

### 2.2 Baseline parameters

We collected 25 females from Trout Pond, a location in which *H. formosa* experience high predation rates and, consequently, high per capita food levels in adults (Leips & Travis, 1999; MacRae & Travis, 2014) We collected fish in mid‐May, which is the peek breeding season, to quantify the reproductive parameters of females in the field as a baseline against which to assess the effects of our experimental manipulations and the realism of the results. We collected females with dip nets and held them for 48 hr in a large stock aquarium (76 l). We collected newborn offspring produced in this period to assess offspring size at parturition. After 48 hr, we euthanized all females in MS‐222 and preserved them in formalin. We measured female standard length (SL), and from each female, tallied the number of embryos in the ovary and their developmental stages, using a modified version of Reznick's (1981) classification system: early‐eyed, mid‐eyed, late‐eyed, and very late‐eyed stages. We freeze‐dried the neonates from the aquarium and weighed them to the nearest 0.001 mg.

### 2.3 Experiment 1

On the 28th of February, about 100 adult female *H. formosa* were caught by dip net in Trout Pond and Moore Lake in Tallahassee, FL. Moore Lake is ecologically similar to Trout Pond and *H. formosa* in Moore Lake have comparable densities and life histories to those in Trout Pond. Twenty 19 L glass aquaria tanks were filled with well water and set up with an airline tube system going into each tank. The experimental tanks were kept in a temperature controlled room (22°C) on a 14:10 light:dark cycle under aquarium lamps at Florida State University. Two‐thirds of the water in each tank was replaced with fresh well water three times a week, and the tanks were cleaned bi‐weekly. Every other week a tank was randomly tested for appropriate pH, ammonia, and nitrate levels.

This experiment was a factorial design in which density level (low or high) was crossed with food level per aquarium (low or high). The aquarium tanks were set up side by side and alternated by high and low density populations. In the low‐density condition, there were two females and one male, and the high‐density condition contained eight females and two males. While the sex ratios differ between density treatments (which was a consequence of male rarity at the time of collection), it is unlikely that this had a major effect on the results. Sex ratios in nature are heavily female biased (Leips & Travis, 1999; Richardson, Gunzburger, & Travis, 2006), and males feed at very low rates (Travis et al., 1987). In the low‐food treatment, each tank received 10 mg daily; in the high‐food treatment, each tank received 50 mg daily. We fed fish ground TetraMin flake food, a diet on which the species expresses values of life‐history traits comparable to those observed in natural populations (Hale & Travis, 2015; Travis et al., 1987). The 20 tanks were set up side by side and alternated by high‐ and low‐population density environments. Trout Pond *H. formosa* were used in eight of the tanks, and Moore Lake *H. formosa* were used for the remaining 12 tanks.

For 5 weeks following the first four, we collected offspring daily and recorded the number of offspring per tank. Newborn offspring were euthanized with MS‐222, preserved in formalin, and later freeze‐dried and weighed to the nearest 0.001 mg. The study was conducted for a total of 9 weeks. The replacement females were as close in size as possible to the females already in the tank, within 2–3 mm standard length. One low density/high food replicate tank from Trout Pond was omitted from the study because the male and both females died after the first week. Throughout the rest of the experiment there were two dead females, both from separate high density/high food populations, and one dead male from a low food/high density population.

At the end of the 9 weeks, we euthanized the females in MS‐222 and stored them in formalin, the males were placed back in their respective stock tanks. We measured each female's standard length to the nearest 0.01 mm and then dissected her to count the number of embryos in her ovary and assigned each embryo to one of four developmental stages, as we did for the baseline data.

### 2.4 Experiment 2

On the 25th of May, about 100 adult female and 50 adult male *H. formosa* were caught by dip net at Trout Pond in Tallahassee, FL. Laboratory conditions were set up the same as experiment one. The 20 tanks were set up side by side and alternated by high and low density environments and in food levels. There were four environmental conditions total: high density/high food, low density/low food, high density/low food, and low density/low food. The females placed in each tank were selected such that they were relatively the same size per tank. In the low‐density condition, there were two females and two males; in the high‐density condition, there were eight females and three males. We used more males than in Experiment 1 in order to provoke more mating activity and higher reproductive rates. Aquaria selected for a high food diet were fed 20 mg of ground TetraMin per female every day (160 mg total ration in high density/high food, 40 mg total ration in low density/high food treatments). Those with a low food diet were given 5 mg of TetraMin per female every day (40 mg total ration in the high density/low food, and 10 mg total ration in the low density/low food treatments). These levels of daily per capita food are within the range produced in Experiment 1. The lower daily per capita food level in this experiment, 5 mg, is the same as the per capita food level produced in the low food/low density treatment of Experiment 1; the higher daily level (20 mg), is slightly less than the 25 mg level produced in Experiment 1 in its high food/low density treatment. As in Experiment 1, we retained a small number of leftover fish in a stock tank to serve as replacements for any females that died throughout the experiment to maintain the density treatments. Overall, three adult females died throughout the experiment, two from high density populations and one from a low density population. No males died during the second experiment. For the 5 weeks following the first month, we collected offspring daily and followed the same procedures that we used in Experiment 1.

### 2.5 Statistical analyses

We used general linear models for analyses of per capita offspring production rate (number of offspring collected divided by number of females in the aquarium) and general linear mixed models for analyses of neonate mass, total number of embryos found in females, and mass of very late‐eyed embryos. In both sets of analyses, food, density, and, in Experiment 1, population were fixed effects. The per capita offspring production rate is a characteristic of each replicate aquarium within a treatment combination. The other variables are characteristics of individuals (females or neonates) found within each aquarium. For these variables, we used the identity of the individual replicate aquarium within each treatment combination as a random effect in the general linear mixed model. We began each analysis with all main effects and two‐way interactions and used backward elimination to arrive at a final model. At each step, we dropped any two‐way interaction that was not significant and whose *F*‐value was below 1.0. We examined residuals to make sure that they satisfied assumptions of normal‐theory analyses. Data on offspring production rate in Experiment 1 were skewed, with two outliers. We analyzed the data with and without these aquaria; the results were similar but the analyses without these aquaria produced residuals that were in accord with normal‐theory analyses, so we present these results in the text.

The level of superfetation was a categorical response, with females carrying embryos in 1–4 stages. To analyze this response, we used a log‐linear model of the counts of females from different food or density levels that fell into one of the categories of superfetation. Because of the small sample size for a 2 × 2 × 4 classification, we combined stages 1 and 2 into a single stage and stages 3 and 4 into a single stage. We analyzed each association of food level and density level with the level of superfetation with the Mantel‐Haenszel statistic, which examines the association of food level (or density level) with level of superfetation, holding any effects of density level (or food level) constant. These contingency analyses are the basis for our inferences, although we present average superfetation levels for ease of interpretation. We conducted all analyses with SYSTAT v. 12 (SYSTAT 2007).

## 3 RESULTS

### 3.1 Baseline parameters

Females collected from Trout Pond ranged in size from 15 to 26 mm SL (average 20.96 ± 0.76 *SE*) and carried from 2–38 embryos (average 16.9 ± 1.7). Longer females carried more embryos (*r* = 0.87, *p* < 0.001, *n* = 25). Females displayed a high level of superfetation; the number of embryonic stages represented by a female's developing offspring ranged from 1–4 and, on average, included 3 of the 4 possible stages (average number of stages 3.08 ± 0.13). The average mass of newborn offspring was 0.86 ± 0.03 mg (*n* = 14), a value higher than those reported in previous collections from this population (Leips & Travis, 1999; Schrader & Travis, 2012).

### 3.2 Experiment 1

**Figure 2:**
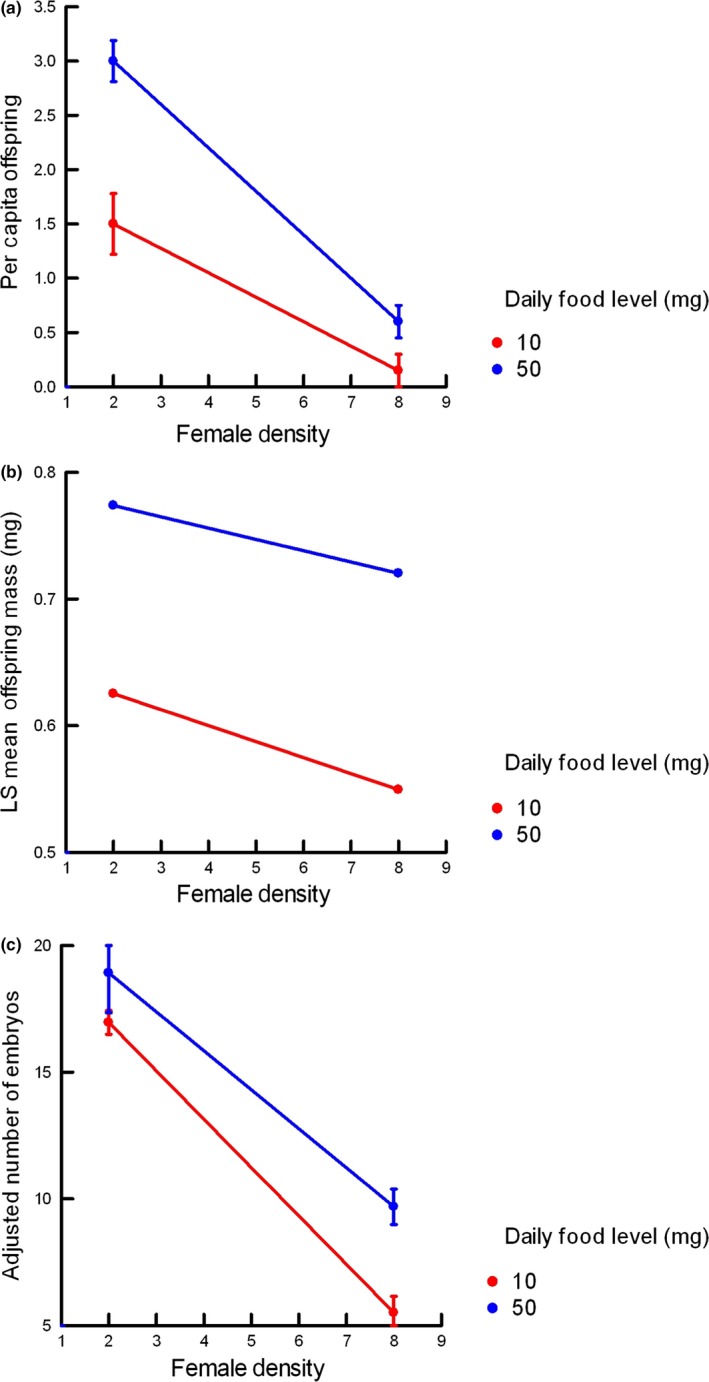
Average offspring production rate, offspring mass, and number of remaining embryos from Experiment 1. (a) Average per capita offspring production rate in Experiment 1 in month 2. (b) Least squares mean offspring mass during month 2 in Experiment 1, based on mixed model results. (c) Least squares means of average number of embryos remaining in females, adjusted for body length, from Experiment 1

Both food and density had strong effects on per capita offspring production rates (Figure 2a). The higher food level increased average offspring production by over threefold at the lower density and almost fourfold at the higher density. Decreasing female density from 8 to 2 increased average offspring production over fourfold. Females from Trout Pond produced more offspring per capita, on average (1.58 ± 0.65 *SE*), than those from Moore Lake (0.80 ± 0.65). Only in the low density/high food combination did the average offspring production rate exceed replacement.

The differences in per capita offspring production rates between the food and density levels were statistically significant (food: *F*_1,11_ = 31.69, *p* < 0.0005; density *F*_1,11_ = 71.05, *p* < 0.0001). Production rates also differed between the populations (*F*_1,11_ = 21.39, *p* < 0.001). The differences between populations did not depend on the level of food (*F*_1,10_ = 0.17) but did depend upon the level of density (*F*_1,11_ = 19.28, *p* < 002). Females from each population had similar production rates at the higher density (ML: 0.35 ± 0.25; TP: 0.41 ± 0.30) but, at the lower density, females from TP produced offspring at a higher rate than females from ML (ML: 1.25 ± 0.25; TP: 3.40 ± 0.36).

There was a significant interaction between food and density levels (*F*_1,11_ = 9.19, *p* < 0.02). Increasing female density depressed offspring production rate much more at the higher food level than at the lower one (Figure 2a). Average offspring production rate was not a strictly linear function of the realized per capita food level in a treatment (total food level divided by density). The average production rate at the low density/low food combination was 25% higher than that at the high density/high food combination (Figure 2a), even though the latter combination should have provided 25% more food per capita. The interaction also made it impossible to determine the relative importance of variation in density level compared to that in food level.

The average mass of newborn offspring at the lower food levels was lower than the baseline levels but comparable to values previously reported from collections in both populations (Figure 2b; Leips & Travis, 1999; Schrader & Travis, 2012). The offspring mass at the higher food level was greater than typical field values for these populations, especially the lower density. Increasing the food level had a greater effect on average offspring mass than decreasing the density; the average offspring mass was about 25% greater at the higher food level at both densities but only 10%–15% greater at the lower density at both food levels.

The general linear mixed model of offspring mass confirmed the predominance of an effect of food level variation over that of density variation. The effect of food level variation was quite significant (*F*_1,33_ = 9.68, *p* < 0.004), while the effect of variation between densities was not (*F*_1,33_ = 1.59). There was no statistical interaction between food and density (*F*_1,33_ = 0.05).

The depressant effect of density on reproductive capacity was evident in the number of embryos remaining in females at the end of the experiment. The number of embryos was correlated with female SL (*r* = 0.60, *p* < 0.001, *n* = 98); so we tested effects of food and density after adjusting for SL via covariance analysis, using the identity of the individual aquaria as a random effect. Females from the higher density carried only half as many embryos as females from the lower density (Figure 2c: main effect of density *F*_1,78_ = 37.24, *p* < 0.0001). While females from the lower food level carried fewer embryos than females from the higher food level at each individual density level, this variation was small and not significant (*F*_1,78_ = 2.66). It is possible that a larger sample size would have detected an effect of food level variation at the higher density and an interaction between food and density levels. The number of remaining embryos was not a function of the realized per capita food levels; females in the high density/high food treatment were carrying about 40% fewer embryos than females in the low density/low food treatment, despite experiencing a 25% higher level of realized per capita food.

The level of superfetation, as measured by the number of stages represented in the embryos within each female, was lower at the higher density (Table 1). At the lower density, all females had embryos in either 3 or 4 stages, whereas at the higher density, females were more likely to have had embryos in only 1 or 2 stages. There was a significant association of density level and level of superfetation (*χ* ^2^ = 8.80, *df* = 1, *p* < 0.005). For comparison, the average number of stages found in a female at the lower density (3.61 ± 0.12) exceeded the average for females at the higher density (2.72 ± 0.12) and the average for the baseline females collected from Trout Pond (3.08 ± 0.13).

**Table 1:** Numbers of females carrying embryos representing from 1 to 4 developmental stages as a function of food and density levels in each experiment

Experiment	Treatment	Level	Number of embryonic stages			
			1	2	3	4
1	Density	2 Females	0	0	7	11
		8 Females	11	16	26	18
	Food	10 mg	7	11	18	9
		50 mg	4	5	15	20
2	Density	2 Females	0	2	10	4
		8 Females	3	10	35	10
	Food	5 mg	1	7	24	5
		20 mg	2	5	21	9

While females had embryos in stages 1 to 4 at both food levels, females from the higher food level were more likely to have embryos in 3 or 4 stages. There was a weak but significant association of food level with level of superfetation (*χ* ^2^ = 4.14, *df* = 1, *p* < 0.05), which suggests that the effect of food level on superfetation was weaker than that of density. Females from the lower food level displayed an average level of superfetation (2.64 ± 0.15) well below that observed in the baseline females from Trout Pond, whereas females from the higher food level displayed a level of superfetation (3.16 ± 0.14) comparable to that seen in the baseline females.

### 3.3 Experiment 2

**Figure 3:**
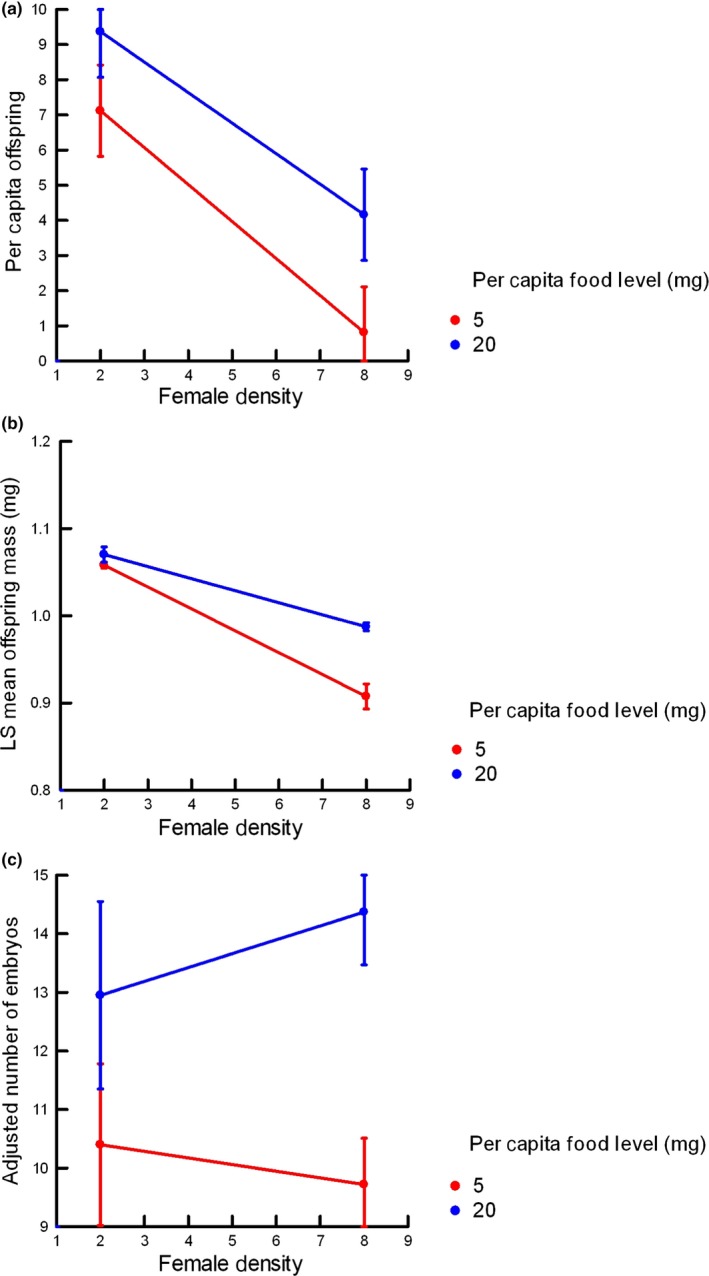
Average offspring production rate, offspring mass, and number of remaining embryos from Experiment 2. (a) Average per capita offspring production rate in Experiment 2 in month 2. (b) Least squares mean offspring mass during month 2 in Experiment 2, based on mixed model results. (c) Least squares means of average number of embryos remaining in females, adjusted for body length, from Experiment 2

As in experiment 1, decreasing the food level or increasing the density reduced the per capita offspring production rate but, in this case, the effect of increasing density was much greater (Figure 3a). This distinction was reflected in the statistical analysis; both food level variation (*F*_1,12_ = 4.60, *p* = 0.05) and density level variation (*F*_1,12_ = 19.53, *p* < 0.001) were significant, but the fourfold variation in density created an effect size (5.27) that was over twice that created by the fourfold variation in per capita food level (2.55). There was no significant interaction between the two experimental factors (*F*_1,12_ = 0.17).

The average mass of newborn offspring in all treatment combinations was much higher than baseline levels and higher than the values from prior field collections from this population (see Figure 3b). While the pattern of the data is similar to that in Experiment 1 (smaller offspring at lower food levels and higher densities), the analytical results are quite different. Whereas in Experiment 1, there was no significant effect of density, in Experiment 2 there was such an effect, although not one that was strong (*F*_1,227_ = 4.19, *p* < 0.04). In a similarly contrarian vein, whereas, in Experiment 1, food level was a strong influence on offspring mass, in this experiment it was not significant (*F*_1,227_ = 0.40). The changes in offspring mass, even when significant, were smaller than those in Experiment 1, on the order of 10%. There was no evidence of any statistical interaction between food and density levels (*F*_1,227_ = 0.34).

As in Experiment 1, the number of embryos remaining in a female was correlated with her SL (*r* = 0.59, *p* < 0.001, *n* = 77), so we tested effects of food and density after adjusting for SL via covariance analysis, using individual aquarium as a random effect, as in Experiment 1. Unlike the results for per capita offspring production, after adjusting for SL, there were no detectable effects of either density (*F*_1,58_ = 0.70) or the interaction of density and food levels (*F*_1,58_ = 0.05). The best model included SL and food level; however, in that model the effect of food level, adjusted for female SL, was not significant (Figure 3c; *F*_1,60_ = 3.05, *p* ≅ 0.08). Given the *F*‐ and *p*‐values for food level, it is possible that a larger experiment would have found that the higher food level was associated with a greater number of embryos, after adjusting for female length.

Unlike in Experiment 1, there was no detectable statistical effect of either food‐ or density‐level variation on the level of superfetation (Table 1). The trends in the level of superfetation among females from different density and food levels were the same as those in Experiment 1, but any undetected effects of the experimental treatments were certainly much smaller than those in Experiment 1. The average level of superfetation among these females was 2.94 ± 0.08, a level comparable to that seen in females in Trout Pond and intermediate between the levels observed in females at lower and higher densities in Experiment 1.

## 4 DISCUSSION

Variation in social density and per capita food acted independently of each other on reproductive parameters in the Least Killifish. This conclusion was not evident in Experiment 1, in which density and food level interacted significantly on reproductive rate. An interaction implies that the effects of each factor cannot be described independently of the effects of the other. The design of this experiment, which varied total food availability and density, confounded variation in social density with variation in per capita food levels, making it impossible to discern the relative strength of each factor.

The results from Experiment 1 could mislead one into concluding that only the realized per capita food levels (obtained by dividing total food by density) mattered, regardless of the specific combination of total food and density that produced them (Wilbur, 1977b). The average values of offspring mass and, to a large extent, reproductive rate followed the rank order of the realized per capita food levels; higher per capita levels produced much greater values of both variables and slightly different per capita food levels (low density/low food and high density/high food) produced slightly different values. However, examination of the number of embryos remaining in females suggested something different. The number of remaining embryos was noticeably and significantly greater in the two low density treatment combinations, despite their having a fourfold difference in realized per capita food levels (Figure 2c). In addition, two combinations with similar realized per capita food levels (low density/low food and high density/high food) had very different embryo counts. A similar pattern was evident in levels of superfetation (Borg et al., 2006).

There were no interactions between food levels and density in Experiment 1 on average offspring mass and the number of remaining embryos. Average offspring mass was not significantly affected by density but was strongly affected by food level. The relative importance of these factors was reversed in the average number of remaining embryos; density played a stronger role than food level, consistent with the strong role of density on offspring reproductive rate.

The results of Experiment 2, in which we manipulated per capita food levels and social density, indicated that omit food level and social density acted independently. There were no interactions between food and social density levels for any variable, and none of the statistical tests were close to significance. In addition, the results of this experiment indicated that variation in social density exerted a stronger control on reproductive rate (per capita production and number of remaining embryos) than variation in food level, whereas food level variation exerted a stronger influence on average offspring mass. The results on number of remaining embryos and average offspring were consistent with those from Experiment 1. Given the lack of any statistical interaction between food and density in Experiment 1, we conclude that, in general, food level variation was the stronger influence on offspring mass and social density was the stronger influence on reproductive rate.

Increased food level and decreased density were associated with increased offspring production rate and higher levels of superfetation, although the effects on superfetation were only statistically significant in Experiment 1. These results are consistent with prior work on *H. formosa* (Leips et al., 2000; Travis et al., 1987) in which higher levels of superfetation were associated with higher offspring production rates. Our results suggest that superfetation might enable female *H. formosa* to accelerate the gestation period under favorable conditions and thereby accelerate population growth. Theory (Trexler & DeAngelis, 2010) and empirical data (Pollux & Reznick, 2011) suggest that matrotrophy and superfetation are advantageous only in stable environments, but the possibility that superfetation might enable more rapid gestation has not been considered.

The strong compensatory effect of social density reinforces the results of previous studies in a variety of organisms, in which the effects of density variation were examined while holding per capita food levels constant at a single value or when individuals were fed ad libitum (Boonstra & Boag, 1992; Borg et al., 2006; Kuznetsov, Tchabovsky, Kolosova, & Moshkin, 2004; Ramsay et al., 2006; Rodd, Reznick, & Sokolowski, 1997; Viblanc, Claire, Nelly, René, & Daniel, 2014; Weber et al., 2011). There are several mechanisms through which this can occur, for example, social dominance interactions (Borg et al., 2006), chemical inhibition (Burns, 1995; Lutnesky & Adkins, 2003), or pathogen transmission (Steinwascher, 1979). The discrepancy between the rate of offspring production and the number of offspring found in female ovaries, especially in Experiment 1, is similar to the results described by Lutnesky and Adkins (2003) and Borg et al. (2006). In each study, increased crowding affected female ovary size and numbers of mature ova.

The interpretation that density effects are stronger than food level effects in Experiment 2 must be tempered. For one reason, social interactions can change markedly with changes in experimental conditions; smaller arenas may promote increased aggression at rates not seen in natural populations. For another, a conclusion of this type may be specific to the precise levels of density and food used in the experiment. Had we used wider variation in food levels, we may have seen a greater role for food level variation. If anything, our variation in density was less than the variation seen among populations (Leips & Travis, 1999; MacRae & Travis, 2014; Richardson et al., 2006) and so, were we to extrapolate to natural conditions, we may have underestimated the strength of the effect of social density.

Regardless of whether these experiments under‐ or overestimated the relative importance of social density, they demonstrated that social density exerts a substantial, measurable effect on reproduction, independently of the effects food level. Interactive effects between density and resource levels, or other environmental conditions that affect energetic demand, play large roles in a variety of ecological issues, including population regulation (Dennis & Otten, 2000; Fowler & Pease, 2010; Gamelon et al., 2017; Spanio, Hidalgo, & Munoz, 2017), pairwise species interactions (Dallalio, Brand, & Grant, 2017; Dunson & Travis, 1991), and theories for the role of competition in structuring multi‐species assemblages (Grime & Pierce, 2012; Tilman, 1988). The extent to which these interactions reflect changes in the balance between nutrient acquisition and metabolic demand has only just begun to be explored (Ghedini, White, & Marshall, 2017). The results reported here indicate that understanding the source of those interactions could be a major line of inquiry in understanding how competition molds the ecology and evolution of animal populations.

## CONFLICT OF INTEREST

None declared.

## AUTHOR CONTRIBUTIONS

JT originally came up with the idea. KL and JT developed the experimental design. JT, KL, and others completed the fieldwork. KL performed the experiments, and JT analyzed the data. JT wrote the manuscript; KL wrote the methods and completed the references. JT and KL reviewed the manuscript and edited as needed.

## DATA ACCESSIBILITY

All offspring and embryo data will be stored in Dryad upon publication.

## References

[ece34634-bib-0001] Bassar, R. D. , Ferriere, R. , López‐Sepulcre, A. , Marshall, M. C. , Travis, J. , Pringle, C. M. , & Reznick, D. N. (2012). Direct and indirect ecosystem effects of evolutionary adaptation in the Trinidadian guppy (*Poecilia reticulata*). American Naturalist, 180, 167–185. 10.1086/666611 22766929

[ece34634-bib-0002] Batzli, G. O. (1977). Population dynamics of the white‐footed mouse in floodplain and upland forests. The American Midland Naturalist, 97, 18–32. 10.2307/2424681.

[ece34634-bib-0003] Berven, K. A. , & Chadra, B. G. (1988). The relationship among egg size, density and food level on larval development in the wood frog (*Rana sylvatica*). Oecologia, 75, 67–72. 10.1007/BF00378815 28311835

[ece34634-bib-0004] Beserra, E. B. , Freitas, E. M. , Souza, J. T. , Fernandes, C. R. M. , & Santos, K. D. (2009). Ciclo de vida de Aedes (Stegomyia) aegypti (Diptera, Culicidae) em águas com diferentes características. Iheringia. Série Zoologia, 99, 281–285. 10.1590/S0073-47212009000300008

[ece34634-bib-0005] Boonstra, R. , & Boag, P. T. (1992). Spring declines in *Microtus pennsylvanicus* and the role of steroid hormones. Journal of Animal Ecology, 61, 339–352. 10.2307/5326

[ece34634-bib-0006] Boonstra, R. , & Krebs, C. J. (2012). Population dynamics of red‐backed voles (Myodes) in North America. Oecologia, 168, 601–620. 10.1007/s00442-011-2120-z 21947547

[ece34634-bib-0007] Borg, J. A. , Rowden, A. A. , Attrill, M. J. , Schembri, P. J. , & Jones, M. B. (2006). Wanted dead or alive: High diversity of macroinvertebrates associated with living and ‘dead’ *Posidonia oceanica* matte. Marine Biology, 149, 667–677. 10.1007/s00227-006-0250-3

[ece34634-bib-0008] Brazill‐Boast, J. , Pryke, S. , & Griffith, S. C. (2013). Provisioning habitat with custom‐designed nest‐boxes increases reproductive success in an endangered finch. Austral Ecology, 38, 405–412. 10.1111/j.1442-9993.2012.02424.x

[ece34634-bib-0009] Brent, C. S. (2010). Stage‐specific effects of population density on the development and fertility of the Western Tarnished Plant Bug, *Lygus hesperus* . Journal of Insect Science, 10, 49 10.1673/031.010.4901 20572784 PMC3014799

[ece34634-bib-0010] Burns, C. W. (1995). Effects of crowding and different food levels on growth and reproductive investment of Daphnia. Oecologia, 101, 234–244. 10.1007/BF00317289 28306796

[ece34634-bib-0011] Carvalho, G. R. , & Hughes, R. N. (1983). The effect of food availability, female culture‐density and photoperiod on ephippia production in *Daphnia magna* Straus (Crustacea: Cladocera). Freshwater Biology, 13, 37–46. 10.1111/j.1365-2427.1983.tb00655.x

[ece34634-bib-0012] Dallalio, E. A. , Brand, A. B. , & Grant, E. H. C. (2017). Climate‐mediated competition in a high‐elevation salamander community. Journal of Herpetology, 51, 190196 10.1670/15-157

[ece34634-bib-0013] Dash, M. C. , & Hota, A. K. (1980). Density effects on the survival, growth rate, and metamorphosis of *Rana tigrina* tadpoles. Ecology, 61, 1025–1028. 10.2307/1936818

[ece34634-bib-0014] Dennis, B. , & Otten, M. R. M. (2000). Joint effects of density dependence and rainfall on abundance of San Joaquin kit fox. The Journal of Wildlife Management, 64, 388–400. 10.2307/3803237

[ece34634-bib-0015] Dettmer, A. M. , Novak, M. A. , Meyer, J. S. , & Suomi, S. J. (2014). Population density‐dependent hair cortisol concentrations in rhesus monkeys (*Macaca mulatta*). Psychoneuroendocrinology, 42, 59–67. 10.1016/j.psyneuen.2014.01.002 24636502 PMC3959662

[ece34634-bib-0016] Doidge, D. W. , Croxall, J. P. , & Baker, J. R. (1984). Density‐dependent pup mortality in the Antarctic fur seal *Arctocephalus gazellu* at South Georgia. Journal of Zoology, 202, 449–460. 10.1111/j.1469-7998.1984.tb05095.x

[ece34634-bib-0017] Dunson, W. A. , & Travis, J. (1991). The role of abiotic factors in community organization. The American Naturalist, 138, 1067–1091. 10.1086/285270

[ece34634-bib-0018] Forrester, G. , Harmon, L. , Helyer, J. , Holden, W. , & Karis, R. (2011). Experimental evidence for density‐dependent reproductive output in a coral reef fish. Population Ecology, 53, 155–163. 10.1007/s10144-010-0225-6

[ece34634-bib-0019] Fowler, N. L. , & Pease, C. M. (2010). Temporal variation in the carrying capacity of a perennial grass population. The American Naturalist, 175, 504–512. 10.1086/651592 20302423

[ece34634-bib-0020] Gamelon, M. , Focardi, S. , Baubet, E. , Brandt, S. , Franzetti, B. , Ronchi, F. , … Gaillard, J.‐M. (2017). Reproductive allocation in pulsed‐resource environments: A comparative study in two populations of wild boar. Oecologia, 183, 1065–1076. 10.1007/s00442-017-3821-8 28154966

[ece34634-bib-0021] Gauthier, G. , Berteaux, D. , Krebs, C. , Reid , D. (2009). Arctic lemmings are not simply food limited‐a comment on Oksanen et al. Evolutionary Ecology Research, 11, 483–484.

[ece34634-bib-0022] Ghedini, G. , White, C. R. , & Marshall, D. J. (2017). Does energy flux predict density‐dependence? An empirical field test. Ecology, 98, 3116–3126. 10.1002/ecy.2033 28950411

[ece34634-bib-0023] Grime, J. , & Pierce, S. (2012). The evolutionary strategies that shape ecosystems. Hoboken, NJ: Wiley‐Blackwell.

[ece34634-bib-0024] Guisande, C. (1993). Reproductive strategy as population density varies in *Daphnia magna* (Cladocera). Freshwater Biology, 29, 463–467. 10.1111/j.1365-2427.1993.tb00780.x

[ece34634-bib-0025] Hale, R. E. , & Travis, J. (2015). Effects of water chemistry on the life history of the Least Killifish *Heterandria formosa* and the absence of evidence for local adaptation. Copeia, 103, 51–57. 10.1643/CE-14-042

[ece34634-bib-0026] Herrando‐Pérez, S. , Delean, S. , Brook, B. W. , & Bradshaw, C. J. A. (2012). Density dependence: An ecological Tower of Babel. Oecologia, 170, 585–603. 10.1007/s00442-012-2347-3 22648068

[ece34634-bib-0027] Hooper, H. L. , Sibly, R. M. , Hutchinson, T. H. , & Maund, S. J. (2003). The influence of larval density, food availability and habitat longevity on the life history and population growth rate of the midge *Chironomus riparius* . Oikos, 102, 515–524. 10.1034/j.1600-0706.2003.12536.x

[ece34634-bib-0028] Kolb, A. , Dahlgren, J. P. , & Ehrlén, J. (2010). Population size affects vital rates but not population growth rate of a perennial plant. Ecology, 91, 3210–3217. 10.1890/09-2207.1 21141182

[ece34634-bib-0029] Korpimäki, E. (1993). Does nest‐hole quality, poor breeding success or food depletion drive the breeding dispersal of Tengmalm's owls? Journal of Animal Ecology, 62, 606–613. 10.2307/5382

[ece34634-bib-0030] Krebs, C. J. (2011). Of lemmings and snowshoe hares: the ecology of northern Canada. Proceedings of the Royal Society B: Biological Sciences, 278, 481–489. 10.1098/rspb.2010.1992 PMC3025691 20980307

[ece34634-bib-0031] Krebs, C. J. , Lambin, X. , & Wolff, J. O. (2007). Social behavior and self‐regulation in murid rodents In Wolff J. O., & Sherman P. W. (Eds.), Rodent societies: An ecological and evolutionary perspective (pp. 173–181). Chicago, IL: University of Chicago Press.

[ece34634-bib-0032] Kuznetsov, V. A. , Tchabovsky, A. V. , Kolosova, I. E. , & Moshkin, M. P. (2004). Effect of habitat type and population density on the stress level of midday gerbils (*Meriones meridianus* Pall.) in free‐living populations. Biology Bulletin of the Russian Academy of Sciences, 31, 628–632. BIBU.0000049736.02912.e2 15615454

[ece34634-bib-0033] Laufer, G. , & Maneyro, R. (2008). Experimental test of intraspecific competition mechanisms among tadpoles of *Leptodactylus ocellatus* (Anura: Leptodactylidae). Zoological Science, 25, 286–290. 10.2108/zsj.25.286 18393565

[ece34634-bib-0034] Leips, J. , Richardson, J. M. L. , Rodd, F. H. , & Travis, J. (2009). Adaptive maternal adjustments of offspring size in response to conspecific density in two populations of the least killifish, *Heterandria formosa* . Evolution, 63, 1341–1347. 19425199 10.1111/j.1558-5646.2009.00631.x

[ece34634-bib-0035] Leips, J. , & Travis, J. (1999). The comparative expression of life‐history traits and its relationship to the numerical dynamics of four populations of the least killifish. Journal of Animal Ecology, 68, 595–616. 10.1046/j.1365-2656.1999.00311.x

[ece34634-bib-0036] Leips, J. , Travis, J. , & Rodd, H. F. (2000). Genetic influences on experimental population dynamics of the least killifish. Ecological Monographs, 70, 289–309. 10.1890/0012-9615(2000)070[0289:GIOEPD]2.0.CO;2

[ece34634-bib-0037] Li, X. , Dong, S. , Lei, Y. , & Li, Y. (2007). The effect of stocking density of Chinese mitten crab *Eriocheir sinensis* on rice and crab seed yields in rice–crab culture systems. Aquaculture, 273, 487–493. 10.1016/j.aquaculture.2007.10.028

[ece34634-bib-0038] Lomnicki, A. (1988). Population ecology of individuals. Princeton, NJ: Princeton University Press.

[ece34634-bib-0039] Lutnesky, M. M. F. , & Adkins, J. W. (2003). Putative chemical inhibition of development by conspecifics in mosquitofish, *Gambusia affinis* . Environmental Biology of Fishes, 66, 181–186. 1023696609963

[ece34634-bib-0040] MacRae, P. S. D. , & Travis, J. (2014). The contribution of abiotic and biotic factors to spatial and temporal variation in population density of the least killifish, *Heterandria formosa* . Environmental Biology of Fishes, 97, 1–12. 10.1007/s10641-013-0117-7

[ece34634-bib-0041] Okamoto, D. , Schmitt, R. J. , Holbrook, S. J. , & Reed, D. C. (2012). Fluctuations in food supply drive recruitment variation in a marine fish. Proceedings of the Royal Society B: Biological Sciences, 279, 4542–4550. 10.1098/rspb.2012.1862 PMC3479736 23015631

[ece34634-bib-0042] Oksanen, T. , Oksanen, L. , Dahlgren, J. , & Olofsson, J. (2008). Arctic lemmings, *Lemmus* spp. and *Dicrostonyx* spp.: Integrating ecological and evolutionary perspectives. Evolutionary Ecology Research, 10, 415–434.

[ece34634-bib-0043] Pinot, A. , Barraquand, F. , Tedesco, E. , Lecoustre, V. , Bretagnolle, V. , & Gauffre, B. (2016). Density‐dependent reproduction causes winter crashes in a common vole population. Population Ecology, 58, 395–405. 10.1007/s10144-016-0552-3

[ece34634-bib-0044] Pollux, B. J. , & Reznick, D. N. (2011). Matrotrophy limits a female's ability to adaptively adjust offspring size and fecundity in fluctuating environments. Functional Ecology, 25, 747–756. 10.1111/j.1365-2435.2011.01831.x

[ece34634-bib-0045] Ramsay, J. M. , Feist, G. W. , Varga, Z. M. , Westerfield, M. , Kent, M. L. , & Schreck, C. B. (2006). Whole‐body cortisol is an indicator of crowding stress in adult zebrafish, *Danio rerio* . Aquaculture, 258, 565–574. 10.1016/j.aquaculture.2006.04.020

[ece34634-bib-0046] Reznick, D. (1981). “Grandfather effects”: The genetics of interpopulation differences in offspring size in the mosquito fish. Evolution, 35, 941–953. 10.1111/j.1558-5646.1981.tb04960.x 28581048

[ece34634-bib-0047] Reznick, D. , Callahan, H. , & Llauredo, R. (1996). Maternal effects on offspring quality in poeciliid fishes. American Zoologist, 36, 147–156. 10.1093/icb/36.2.147

[ece34634-bib-0048] Richardson, J. M. L. , Gunzburger, M. S. , & Travis, J. (2006). Variation in predation pressure as a mechanism underlying differences in numerical abundance between populations of the poeciliid fish *Heterandria formosa* . Oecologia, 147, 596–605. 10.1007/s00442-005-0306-y 16341890

[ece34634-bib-0049] Rodd, H. F. , Reznick, D. N. , & Sokolowski, M. B. (1997). Phenotypic plasticity in the life history traits of guppies: Responses to social environment. Ecology, 78, 419–433. 10.1890/0012-9658(1997)078[0419:PPITLH]2.0.CO;2

[ece34634-bib-0050] Rose, K. E. , Rees, M. , & Grubb, P. J. (2002). Evolution in the real world: Stochastic variation and the determinants of fitness in *Carlina vulgaris* . Evolution, 56, 1416–1430. 10.1111/j.0014-3820.2002.tb01454.x 12206242

[ece34634-bib-0051] Samhouri, J. F. (2009). Food supply influences offspring provisioning but not density‐dependent fecundity in a marine fish. Ecology, 90, 3478–3488. 10.1890/08-1732.1 20120815

[ece34634-bib-0052] Schrader, M. , & Travis, J. (2012). Assessing the roles of population density and predation risk in the evolution of offspring size in populations of a placental fish. Ecology and Evolution, 2, 1480–1490. 10.1002/ece3.255 22957156 PMC3434941

[ece34634-bib-0053] Spanio, T. , Hidalgo, J. , & Munoz, M. A. (2017). Impact of environmental colored noise in single‐species population dynamics. Physical Review E, 96, 042301 10.1103/PhysRevE.96.042301 29347568 PMC7217512

[ece34634-bib-0054] Steinwascher, K. (1979). Competitive interactions among tadpoles: Responses to resource level. Ecology, 60, 1172–1183. 10.2307/1936965

[ece34634-bib-0055] Tilman, D. (1988). Plant strategies and the dynamics and structure of plant communities. Princeton, NJ: Princeton University Press.

[ece34634-bib-0056] Travis, J. , Farr, J. A. , Henrich, S. , & Cheong, R. T. (1987). Testing theories of clutch overlap with the reproductive ecology of *Heterandria Formosa* . Ecology, 68, 611–623. 10.2307/1938466

[ece34634-bib-0057] Treinys, R. , Bergmanis, U. , & Väli, Ü. (2016). Strong territoriality and weak density‐dependent reproduction in Lesser Spotted Eagles *Clanga pomarina* . Ibis, 159, 343–351. 10.1111/ibi.12454

[ece34634-bib-0058] Trexler, J. C. , & DeAngelis, D. L. (2010). Modeling the evolution of complex reproductive adaptations in poeciliid fishes: Matrotrophy and superfetation In Uribe M. C., & Grier H. J. (Eds.), Viviparous Fishes II (pp. 231–240). Homestead, FL: New Life Publications.

[ece34634-bib-0059] Viblanc, V. A. , Claire, S. , Nelly, M. , René, G. , & Daniel, C. (2014). Energetic adjustments in freely breeding‐fasting king penguins: Does colony density matter? Functional Ecology, 28, 621–631. 10.1111/1365-2435.12212

[ece34634-bib-0060] Warren, E. W. (1973). Modification of the response to high density conditions in the guppy, *Poecilia reticulata* (Peters). Journal of Fish Biology, 5, 737–752. 10.1111/j.1095-8649.1973.tb04507.x

[ece34634-bib-0061] Weber, G. M. , Vallejo, R. L. , Lankford, S. E. , Silverstein, J. T. , & Welch, T. J. (2011). Cortisol response to a crowding stress: Heritability and association with disease resistance to *Yersinia ruckeri* in rainbow trout. North American Journal of Aquaculture, 70, 425–433. 10.1577/A07-059.1

[ece34634-bib-0062] Weeks, S. C. (1993). Phenotypic plasticity of life‐history traits in clonal and sexual fish (*Poeciliopsis*) at high and low densities. Oecologia, 93, 307–314. 10.1007/BF00317871 28313428

[ece34634-bib-0063] Wilbur, H. M. (1977a). Density‐dependent aspects of growth and metamorphosis in *Bufo Americanus* . Ecology, 58, 196–200. 10.2307/1935122

[ece34634-bib-0064] Wilbur, H. M. (1977b). Interactions of food level and population density in *rana sylvatica* . Ecology, 58, 206–209. 10.2307/1935124

[ece34634-bib-0065] Wolff, J. O. (1997). Population regulation in mammals: An evolutionary perspective. Journal of Animal Ecology, 66, 1–13. 10.2307/5959

[ece34634-bib-0066] Yang, F. , Hu, G. , Shi, J. J. , & Zhai, B. P. (2015). Effects of larval density and food stress on life‐history traits of *Cnaphalocrocis medinalis* (Lepidoptera: Pyralidae). Journal of Applied Entomology, 139, 370–380. 10.1111/jen.12179

